# LRR-Containing Oncofetal Trophoblast Glycoprotein 5T4 Shapes Neural Circuits in Olfactory and Visual Systems

**DOI:** 10.3389/fnmol.2020.581018

**Published:** 2020-10-28

**Authors:** Akio Tsuboi

**Affiliations:** Graduate School of Frontier Biosciences, Osaka University, Suita, Japan

**Keywords:** LRR-containing membrane protein, 5T4 oncofetal trophoblast glycoprotein, olfactory bulb interneuron, retinal interneuron, odor detection and discrimination

## Abstract

In mammals, the sensory experience can regulate the development of various brain structures, including the cortex, hippocampus, retina, and olfactory bulb (OB). Odor experience-evoked neural activity drives the development of dendrites on excitatory projection neurons in the OB, such as mitral and tufted cells, as well as inhibitory interneurons. OB interneurons are generated continuously in the subventricular zone and differentiate into granule cells (GCs) and periglomerular cells (PGCs). However, it remains unknown what role each type of OB interneuron plays in controlling olfactory behaviors. Recent studies showed that among the various types of OB interneurons, a subtype of GCs expressing oncofetal trophoblast glycoprotein 5T4 is required for simple odor detection and discrimination behaviors. Mouse 5T4 (also known as Tpbg) is a type I membrane glycoprotein whose extracellular domain contains seven leucine-rich repeats (LRRs) sandwiched between characteristic LRR-N and LRR-C regions. Recently, it was found that the developmental expression of *5T4* increases dramatically in the retina just before eye-opening. Single-cell transcriptomics further suggests that 5T4 is involved in the development and maintenance of functional synapses in a subset of retinal interneurons, including rod bipolar cells (RBCs) and amacrine cells (ACs). Collectively, *5T4*, expressed in interneurons of the OB and retina, plays a key role in sensory processing in the olfactory and visual systems.

## Introduction

Cell adhesion molecules with immunoglobulin, cadherin, and leucine-rich repeat (LRR) domains are involved in target recognition in synaptogenesis (Sanes and Zipursky, 2020). In particular, the membrane proteins containing LRR motifs in the extracellular domain organize excitatory and inhibitory synapses by forming binding interfaces for a broad spectrum of interactions. Several mammalian LRR proteins are implicated in synaptic specificity, although in most cases, it remains unclear whether they promote specificity or synaptogenesis. These include three fibronectin LRR transmembrane proteins (FLRTs), four LRR transmembrane neuronal proteins (LRRTMs), six Slit and neurotrophic receptor tyrosine kinase (NTRK)-like family proteins (Slitrks), five synaptic adhesion-like molecules (SALMs), and three netrin-G ligands (NGLs; Schroeder and de Wit, 2018). Most of these bind heterophilically to other proteins, including FLRTs to latrophilins, LRRTMs to neurexins, and NGLs to netrin G1 and G2 (de Wit and Ghosh, 2014). Here, I show a member of the extracellular-type LRR membrane proteins, which is expressed in interneurons of the olfactory bulb (OB) and retina and plays a key role in sensory processing in the olfactory and visual systems.

## LRR-Containing Oncofetal Trophoblast Glycoprotein 5T4

**Figure 1:**
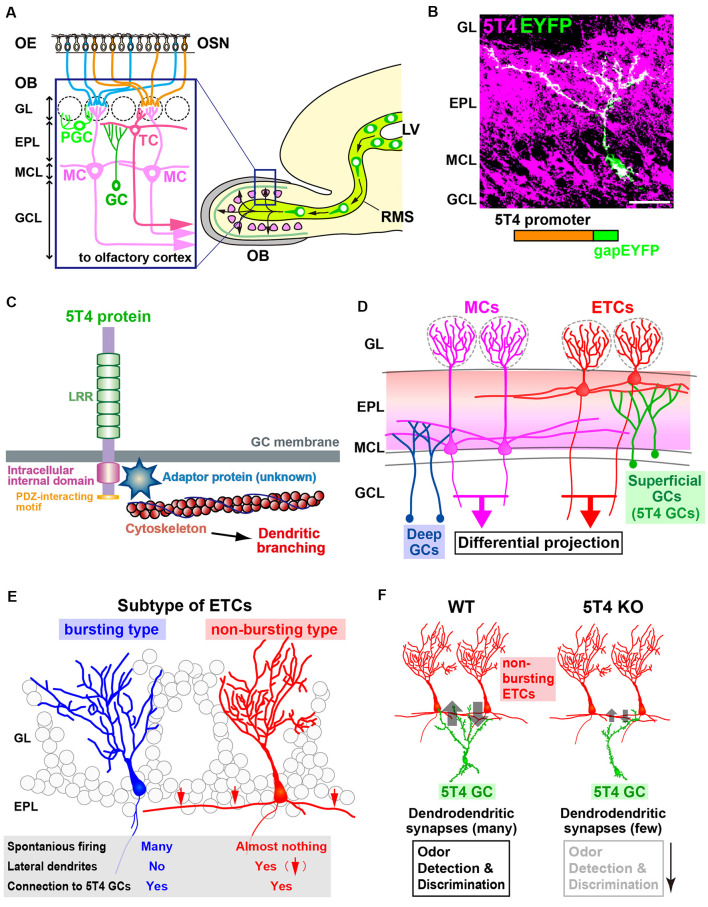
The function of 5T4 in a subtype of granule cells (GCs) in the olfactory bulb (OB). **(A)** Mammalian OB is composed of a distinct laminar structure. A subset of olfactory sensory neurons (OSNs) in the olfactory epithelium (OE) extend their axons to specific glomeruli in the OB. OSN signals activate a specific neural circuit, promoting the dendritic development of inhibitory interneurons through excitatory projection neurons such as tufted cells (TCs) and mitral cells (MCs). OB interneurons are generated continuously in the subventricular zone within the lateral ventricle (LV), migrate through the rostral migratory stream (RMS), and differentiate into inhibitory interneurons such as GCs and periglomerular cells (PGCs). GL, glomerular layer; EPL, external plexiform layer; MCL, mitral cell layer; GCL, granule cell layer. **(B)** Dendritic morphology and laminar location of 5T4 GCs. The lentiviral vector carrying *5T4* promoter-driven *gapEYFP* was injected into the LV of wild-type mice for immunostaining with anti-5T4 and anti-EGFP antibodies. Scale bar, 30 μm. **(C)** Schematic representations of 5T4 protein and 5T4 signaling pathway. The 5T4-intracellular domain, which lacks the PDZ-interacting motif, is necessary for the dendritic morphology of 5T4 GCs (Yoshihara et al., 2012). Recent studies suggest that a member of Ras-like small GTPase superfamily, Rab11, may interact with 5T4 to regulate dendritic branching of 5T4 GCs in the OB (Harris et al., 2018; Siri et al., 2020). **(D)** Schematic drawing of the OB neural circuit. Superficial GCs, including 5T4 GCs, connect preferentially to the lateral dendrites of external TCs (ETCs) at the upper EPL, whereas deep GCs connect mainly to MCs at the deeper EPL. Parallel ETC and MC pathways send distinct odor information *via* their specific routes to different areas in the olfactory cortex. **(E)** ETCs are divided into two different subtypes. Bursting ETCs without lateral dendrites frequently fire the spontaneously, whereas non-bursting ETCs with lateral dendrites do not. 5T4 GCs connect to both bursting and non-bursting ETCs *via* dendrodendritic synapses (Takahashi et al., 2016). **(F)** 5T4 GCs connect to non-bursting ETCs. The dendritic branching of 5T4 GCs is more reduced in *5T4*-knockout (KO) mice (Yoshihara et al., 2012). Notably, GABAergic inputs into non-bursting ETCs are significantly reduced in *5T4*-KO mice, while those into bursting ETCs are unaffected. This gives rise to alterations of olfactory behaviors such as odor detection and discrimination in *5T4*-KO mice.

In the neural circuit of the OB, a family of membrane proteins that localize to specific strata was thought to be implicated in the formation of layer-specific dendrodendritic synaptic connections (Figure 1A; Imamura et al., 2006). Mass spectrometry analyses for membrane proteins in the mouse OB identified 5T4 oncofetal trophoblast glycoprotein (termed 5T4 or Tpbg), a member of the LRR membrane protein family, in interneurons at a specific stratum (Figure 1B). Intriguingly, among the extracellular-type LRR membrane proteins, 5T4 is well conserved in mice (King et al., 1999) and humans (Myers et al., 1994), as well as in nonmammalian species, including CG6959 in the fly (Özkan et al., 2013) and Wnt-activated inhibitory factor 1 (WAIF1) in zebrafish (Kagermeier-Schenk et al., 2011).

5T4 is a type I transmembrane glycoprotein with an N-terminal extracellular domain comprising seven LRRs (24 amino acids each), flanked by characteristic LRR-N and LRR-C regions, and interspersed by seven N-linked glycosylation sites (Figure 1C; King et al., 1999; Imamura et al., 2006; Zhao et al., 2014). The intracellular domain of 5T4 is capped by a class 1 PDZ-interacting motif (Figure 1C; Imamura et al., 2006; Zhao et al., 2014) and contains two serine residues, which are likely phosphorylated by protein kinase Cα (PKCα; Wakeham et al., 2019, 2020).

5T4 was originally identified while searching for molecules with invasive properties that are likely shared by placental trophoblasts and cancer cells (Hole and Stern, 1990). *5T4* is normally expressed at high levels in the brain and ovaries (King et al., 1999; Barrow et al., 2005) and at low levels in other tissues, but is highly expressed in a variety of carcinomas (Southall et al., 1990). Overexpression of *5T4* in murine epithelial cells downregulates E-cadherin, disrupts of cell-to-cell contacts, alters their morphology, and increases their motility (Carsberg et al., 1996). However, *5T4* upregulation is also associated with the differentiation of embryonic stem cells and is essential for epithelial-to-mesenchymal transition (Eastham et al., 2007; Spencer et al., 2007). In embryonic cell lines, 5T4 affects the cytoskeletal organization and cell motility by modulating Wnt/β-catenin signaling (Kagermeier-Schenk et al., 2011). A recent study also reported the expression of *5T4* in epithelial progenitors, such as taste stem and progenitor cells, and taste bud cell precursors, suggesting that it contributes to the maintenance of taste papillae throughout life (Takahashi et al., 2019).

## The Function of 5T4 in a Granule Cell Subtype Within the Olfactory Bulb

Odorants activate specific olfactory sensory neurons (OSNs) expressing the corresponding odorant receptors (Mori and Sakano, 2011). OSN axons project to specific glomeruli in the OB to comprise a specific neural circuit involving glutamatergic excitatory projections of external tufted cells (ETCs) and mitral cells (MCs) that also promote the dendritic development of inhibitory interneurons (Figure 1A; Mori and Sakano, 2011; Lepousez et al., 2013). OB interneurons, such as granule cells (GCs) and periglomerular cells (PGCs), are generated continuously in the subventricular zone within the lateral ventricle (LV) and migrate through the rostral migratory stream (RMS) to the OB, where they differentiate into *γ*-aminobutyric acid (GABA)-releasing inhibitory interneurons (Figure 1A; Imayoshi et al., 2008; Lledo et al., 2008; Whitman and Greer, 2009; Adam and Mizrahi, 2010; Kaneko et al., 2010; Sakamoto et al., 2011). Odor-rich environment and odor deprivation promote and suppress, respectively, dendritic morphogenesis and spinogenesis in newborn OB interneurons (Saghatelyan et al., 2005; Livneh et al., 2014), which are essential for odor detection and discrimination, olfactory learning and memory, and innate olfactory responses including avoidance and sexual behaviors (Breton-Provencher et al., 2009; Sakamoto et al., 2011, 2014; Alonso et al., 2012; Nunes and Kuner, 2015). Cell morphology and lineage analyses revealed that GCs are the largest population of OB interneurons and are subdivided into several subtypes (Orona et al., 1983; Shepherd et al., 2007; Merkle et al., 2014) according to their expression of *calretinin*, Ca^2+^/calmodulin-dependent protein kinase II α subunit (*CaMKIIα*), *5T4*, metabotropic glutamate receptor 2 (*mGluR2*), and *neurogranin* (Imamura et al., 2006; Batista-Brito et al., 2008; Gribaudo et al., 2009; Murata et al., 2011; Merkle et al., 2014; Malvaut et al., 2017). However, the functional specificity that distinguishes each of these GC subtypes in the OB remains unknown, in part because of the paucity of genetically altered mouse lines.

Combinatory screening with DNA microarray and *in situ* hybridization for unilaterally naris-occluded (i.e., odor-deprived) mice revealed that *5T4* expression in OB interneurons is dependent on odor-evoked neural activity (Yoshihara et al., 2012). Imamura et al. (2006) and Yoshihara et al. (2012) observed *5T4* expression in subpopulations of GCs and PGCs, suggesting its role in sensory processing. GCs of the *5T4*-genetic lineage (hereinafter termed 5T4 GCs) in the OB have unique morphological features: their cell bodies are located mostly in the MC layer and some in the inner plexiform layer (IPL) and superficial GC layer; their dendrites ramify in the superficial external plexiform layer (EPL; Figure 1B; Imamura et al., 2006; Yoshihara et al., 2012). *5T4* loss- and gain-of-function experiments *via* knockout (KO) mice and lentiviral vector expression, respectively, demonstrated that 5T4 is necessary and sufficient for dendritic branching of 5T4 GCs in response to odor stimuli (Yoshihara et al., 2012). Takahashi et al. (2016) also used *5T4*-KO and wild-type mice to show that 5T4 GCs play an important role in processing odor information in the OB neural circuit, as described below.

Inhibitory GCs synapse with MCs and ETCs, excitatory projection neurons of the OB (Figure 1D; Mori et al., 1983; Orona et al., 1984). ETCs are further divided into two distinct types: bursting ETCs without lateral dendrites that frequently fire spontaneously and non-bursting ETCs with lateral dendrites that do not fire spontaneously (Figure 1E; Ma and Lowe, 2010). To identify which type synapses with 5T4 GCs, GABA_A_-mediated postsynaptic currents were recorded for individual ETCs after *Channelrhodopsin-2*-expressing 5T4 GCs were stimulated by light (Madisen et al., 2012; Takahashi et al., 2016). These experiments revealed that the apical dendrites of 5T4 GCs form GABAergic synapses with both non-bursting ETCs and bursting ETCs (Figures 1D,E), as well as with MCs. Also, studies on OB slices from *5T4*-KO mice showed that electrode stimulation evoked GABA_A_-mediated postsynaptic currents in bursting ETCs, whereas the currents in non-bursting ETCs were significantly reduced (Figure 1F; Takahashi et al., 2016). As GCs in the OB form reciprocal dendrodendritic synapses with projection neurons (Shepherd et al., 2004), excitatory inputs from ETCs to 5T4 GCs were also examined in wild-type and *5T4*-KO mice. Importantly, the excitatory inputs from ETCs to 5T4 GCs were significantly fewer in *5T4*-KO mice than in the wild type (Figure 1F), consistent with the reduced dendritic branching of *5T4*-deficient GCs (Yoshihara et al., 2012). Taken together, these results demonstrate that 5T4 GCs regulate neural activity in non-bursting ETCs (Takahashi et al., 2016).

The reduced inhibition of non-bursting ETCs combined with the reduced excitation of *5T4*-deficient GCs may affect olfactory behaviors in *5T4*-KO mice (Figure 1F). To assess the physiological role of 5T4 GCs in odor information processing in the OB neural circuit, odor-detection thresholds in wild-type and *5T4*-KO mice were examined by using a habituation-dishabituation test (Takahashi et al., 2016). The sensitivity of odor detection in *5T4*-KO mice was approximately 100-fold lower than in the wild type. Moreover, *5T4*-KO mice were unable to discriminate between two odorants presented simultaneously, although they showed no deficit when the odorants were presented separately in an odor discrimination learning task (Takahashi et al., 2016). Impaired odor discrimination in *5T4*-KO mice was also demonstrated by the prolonged time they spent searching for a buried food pellet when a nonfood-related odorant was presented. Notably, they showed no deficit when searching for the buried food pellet in the absence of the distracting odor. Thus, 5T4 GCs in the OB play a crucial role in both odor detection and discrimination behaviors (Figure 1F; Takahashi et al., 2016, 2018). However, I cannot exclude the possibility that the behavioral changes were due to the elimination of 5T4 PGCs.

Recently, it was reported that zebrafish MCs receive direct interhemispheric projections from their contralateral counterparts, whereas interneurons receive interhemispheric top-down inputs from the contralateral zebrafish homolog of the olfactory cortex (Kermen et al., 2020). Mouse MCs/TCs receive indirect interhemispheric projections from their contralateral counterparts *via* the anterior olfactory nucleus pars externa (Grobman et al., 2018), whereas interneurons receive top-down inputs mostly from the ipsilateral olfactory cortex (Niedworok et al., 2012). The interhemispheric connections of ETCs, whose neural activity is regulated by 5T4 GCs, may enable modulation of odor responses and contribute to the detection of specific odors in a noisy odor background.

## Expression of *5T4* in Rod Bipolar Cells and a Subtype of Amacrine Cells Within the Retina

**Figure 2:**
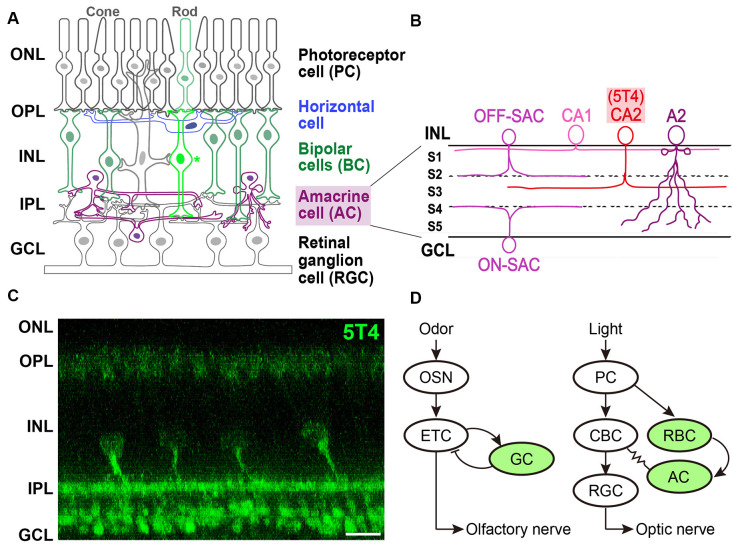
Expression of *5T4* in rod bipolar cells (RBCs) and a subtype of amacrine cells (ACs) in the retina.** (A)** Mammalian retina is composed of a distinct laminar structure. Photoreceptor (rod and cone) cells, interneurons such as horizontal cells, BCs, and ACs; and retinal ganglion cells (RGCs) are drawn schematically. Photoreceptor cells (PCs) form synapses with horizontal cells and BCs at the outer plexiform layer (OPL); BCs and ACs form synapses with each other as well as with RGCs at the inner plexiform layer (IPL). Remarkably, recent single-cell transcriptomics revealed that *5T4* belongs to one of 15 mouse BC clusters (Shekhar et al., 2016) that contains rod BCs (shown as an asterisk), whose processes extend to RGCs in the lower half of the IPL near the ganglion cell layer (GCL; shown in panel **C**), consistent with the previous observations (Imamura et al., 2006; Wakeham et al., 2019, 2020). Axons of RGCs extend through the optic nerve to the brain. ONL, outer nuclear layer; INL, inner nuclear layer. **(B)** Single-cell transcriptomics separated mouse ACs into 63 clusters. Based on the expression of established type-specific markers, several clusters are assigned to known AC types such as starburst ACs (SACs; C17 cluster) and AII (also A2; C3 cluster) ACs (Yan et al., 2020a). Importantly, *5T4* belongs to AC clusters, C25 and C31: glutamate decarboxylase 1 (*Gad1*) is highly expressed in C25 and C31; and tyrosine hydroxylase (*Th*) is highly expressed in C25. These results suggest that 5T4^+^ ACs are GABAergic and catecholaminergic (CAII or CA2) cells, whose dendrites branch at the sublamina (S3) between ON and OFF sublayers in the IPL **(C)**, consistent with the previous observations (Imamura et al., 2006; Wakeham et al., 2019, 2020). Note that the IPL is divided into five sublaminae, S1–S5, with processes of each neuronal type confined to one or a few of them. Panels **(A,B)** are modified from Figure 1 in the article by Yan et al. (2020a), with permission of the journal for use. **(C)** Immunohistochemistry of the adult mouse retina with anti-5T4 antibody (kindly provided by Dr. Keisuke Yonehara at DANDRITE). Scale bar, 10 μm. **(D)** Schematic drawings of neuronal circuits in OB (*left*) and retina (*right*). Green color depicts 5T4-positive cells. (*Left*) Odor stimulation depolarizes OSNs, and connections from OSNs to ETCs produce excitatory outputs. Glutamatergic ETCs are reciprocally connected to GABAergic GCs and receive inhibitory feedback. ETCs extend their axons, forming the olfactory nerve, to the olfactory cortex. (*Right*) Light stimulation hyperpolarizes PCs, which connect to BCs, leading to the production of both ON-type and OFF-type signals. ON-type RGCs receive indirect excitation from rod BCs (RBC), *via* ACs and ON-type cone BCs (CBCs), and direct excitation from the terminals of ON-type CBCs *via* chemical synapses. RGC axons form the optic nerve, which projects to the visual cortex.

Like the OB, the mammalian retina also has a distinct laminar structure (Sanes and Yamagata, 2009). The unique output element is the retinal ganglion cell (RGC) in the ganglion cell layer (Figure 2A). RGC dendrites extend into the IPL and receive inputs from bipolar cells (BCs) and amacrine cells (ACs), the retinal interneurons (Figure 2A; Sterling and Demb, 2004). In the rod pathways of the retina, rod BCs (RBCs) are the first excitatory interneurons in the rod circuit to receive light-dependent synaptic input from rod photoreceptors in the outer plexiform layer (OPL), and give rise to retinal output *via* AII ACs in the IPL (Figure 2A). Although RBCs are primarily responsible for dark-adapted low-light vision, they contribute to retinal output under a diverse range of lighting conditions (Euler et al., 2014); however, the molecular mechanisms involved in RBC adaptation to changing luminance conditions remain unknown (Rampino and Nawy, 2011; Wakeham et al., 2019).

Imamura et al. (2006) identified 5T4-positive interneurons in the adult mouse retina. *5T4* is expressed mainly by RBCs whose processes are dispersed in the lower half of the IPL near the ganglion cell layer, as well as by a subpopulation of ACs whose dendrites branch at a single sublamina between ON and OFF sublayers in the IPL (Figures 2A,C). Recently, Wakeham et al. (2019) performed mass spectroscopy with multiplexed tandem mass tags to reveal that 5T4 acts as a PKCα-dependent phosphoprotein in RBCs. These authors then showed that 5T4 protein localizes to the somas, dendrites, and axon terminals of RBCs, as well as the somas and dendrites of an uncharacterized group of ACs (Figures 2B,C; Wakeham et al., 2020). Interestingly, 5T4 protein is undetectable in the neuron at birth but appears in the putative ACs by postnatal day 6 followed by an expression in RBCs by postnatal day 11. *5T4* expression in RBCs increases remarkably between postnatal days 11 and 12, just before eye-opening, suggesting that it plays a role in the development and maintenance of RBCs and ACs. Possibly, *5T4* expression in RBCs may be induced in response to glutamate-driven spontaneous retinal activity beginning a few days before eye-opening or in response to light-mediated activity, since BCs become light-responsive around postnatal day 10, probably *via* light entering the retina through closed eyelids (Tian and Copenhagen, 2003).

Studies on single-cell transcriptomics have been recently performed to classify neuronal types and reveal the recognition molecules they express (Sanes and Zipursky, 2020). Shekhar et al. (2016) utilized massively parallel single-cell RNA-Seq and optimized computations to reveal 15 clusters of retinal BCs in mice, one of which could be distinguished by its expression of *PKCα* (Puthussery et al., 2010). This rod BC cluster contains more than 100 enriched genes (Figure 2C; Shekhar et al., 2016), including all previously reported RBC markers such as *5T4* (Imamura et al., 2006). Intriguingly, single-cell RNAseq analysis revealed that *5T4* is expressed in another retinal cell type, a subtype of GABAergic ACs (Macosko et al., 2015; Shekhar et al., 2016). Indeed, high-throughput single-cell RNA-Seq analyses (Yan et al., 2020a) identified 63 distinct AC clusters, including those expressing *5T4* (clusters 25 and 31; Figure 2C). Clusters 25 and 31 also contained *Gad1* encoding glutamate decarboxylase 1, suggesting that these ACs are GABAergic, along with *Tac1*, encoding tachykinin precursor 1. Furthermore, the *Th* gene for tyrosine hydroxylase was expressed by ACs in cluster 25, suggesting that 5T4^+^ ACs are also catecholaminergic (CAII or CA2; Figure 2B). These results strongly suggested that *5T4* is expressed not only by excitatory RBCs but also by inhibitory ACs (Figure 2C; Imamura et al., 2006; Wakeham et al., 2019). However, the relative amount of *TPBG* (the *5T4* homolog) mRNA seems to be low in macaque (Peng et al., 2019) or human (Yan et al., 2020b) retina. *TPBG* is expressed in a subtype of RGCs (18 types in macaque or 12 in human), termed ON midget RGCs (Peng et al., 2019; Yan et al., 2020b). Notably, the contrast-response functions of ON midget RGCs have lower thresholds, higher gain, and are more linear than those of OFF midget RGCs in humans (Soto et al., 2020).

## Perspectives of 5T4 Function in the Retinal Neural Circuit

The functional organization of neuronal circuits for signal processing in the OB may be more similar to that in the retina than previously thought (Gollisch and Meister, 2010; Gire et al., 2013). In the OB circuit, odor stimuli depolarize OSNs, which connect to glutamatergic ETCs to produce excitatory outputs. ETCs also receive inhibitory feedback from reciprocal connections with GCs, and project to the olfactory cortex through the olfactory nerve. In the retinal circuit, light stimuli hyperpolarize photoreceptor cells (PCs), which connect to BCs to produce both ON-type and OFF-type bipolar signals. ON-type RGCs receive indirect excitation from rod BCs (RBCs) *via* AII ACs and ON-type cone BCs, and direct excitation from the terminals of ON-type cone BCs *via* chemical synapses (Figure 2D). RGCs, which represent the output layer of the retina, project their axons *via* the optic nerve to the lateral geniculate nucleus and the superior colliculus.

Intriguingly, these observations suggest a similarity between the OB and retinal circuits. In the OB, *5T4* expression is required for odor stimulation-dependent dendritic branching of GCs (Yoshihara et al., 2012) and crucial for odor detection and discrimination behaviors (Figure 1D; Takahashi et al., 2016, 2018). I hypothesize that *5T4* expression by excitatory RBCs and inhibitory ACs in the retina may regulate dendritic branching in response to light stimuli and contribute to dim-light detection and visual pattern discrimination.

In other systems, 5T4 changes Wnt signaling to modulate cytoskeletal rearrangement and cell morphology during embryonic development and cancer progression (Kagermeier-Schenk et al., 2011; Stern et al., 2014). In the OB, *Wnt5a* is expressed in interneurons, and a disruption of this reduces the extension of dendrites from GCs (Pino et al., 2011). Thus, Wnt5a production may regulate the Wnt-signaling pathway to promote the dendritic branching of GCs in the OB (van Amerongen and Nusse, 2009). In the developing mouse retina, Wnt signaling between rods and RBCs is involved in functional synaptic targeting and OPL lamination. In *Wnt5a*-KO mice, RBCs are mistargeted and give rise to the formation of an ectopic OPL (Sarin et al., 2018). This suggests that a Wnt-dependent mechanism is activated during the development of RBC dendrites and axons. Furthermore, *5T4* expression increases concomitantly with the development processes of RBCs that depend on the Wnt-signaling pathway (Wakeham et al., 2019), and regulates Wnt signaling in other embryonic and cancer tissues (Kagermeier-Schenk et al., 2011; Stern et al., 2014). These results further suggest that 5T4 may modulate similar signaling pathways in the retina and OB, and thus play crucial roles in the development and maintenance of neurites of retinal and OB interneurons. Future electrophysiological analyses of *5T4-expressing* RBCs and ACs and behavioral analyses of *5T4*-KO mice will help to elucidate its functional role in visual processing in the retinal neural circuit.

Yoshihara et al. (2012) revealed that the intracellular domain of 5T4 is necessary and sufficient for dendritic branching of 5T4 GCs, based on results of domain deletion and swapping experiments. Further, the 5T4 intracellular domain that lacks the PDZ-interacting motif is crucial (Figure 1C). They attempted to identify the molecules that interact with this domain by a yeast two-hybrid screen but failed. Harris et al. (2018) used a proteomic screen to identify ARF6, Rab18, and Rab11 as interacting proteins that control the expression and distribution of 5T4 in breast cancer cells. Interestingly, loss of *Rab11*, encoding a member of the Ras superfamily of small GTPases prevents the endocytosis of 5T4, resulting in its accumulation in the plasma membrane. Hence, evolutionarily conserved Rab11 may be a critical regulator for the sorting and trafficking of 5T4-containing vesicles to the cytoplasmic membrane. Indeed, Rab11-recycling endosomes are essential for growth cones, synapse architecture regulation, and neuronal migration (Welz et al., 2014). Siri et al. (2020) recently showed that the localization of these endosomes correlates with the developmental stage of hippocampal neurons, and that suppression of *Rab11* expression increases dendritic branching (but not total dendritic length) and results in a misdistribution of dendritic proteins *in vitro* and *in vivo*. The interaction between Rab11-recycling endosomes and 5T4 may be required for proper dendritic branching of 5T4 GCs, thus controlling key aspects of synaptic plasticity (Figure 1C). Future studies on the molecular targets that interact with extracellular and intracellular domains of 5T4 will shed light on its physiological roles in the neural circuitry driving odor- and vision-associated behaviors.

## Author Contributions

AT wrote the article.

## Conflict of Interest

The author declares that the research was conducted in the absence of any commercial or financial relationships that could be construed as a potential conflict of interest.

## Abbreviations

AC: amacrine cell
BC: bipolar cell
CaMKIIα: Ca^2+^/calmodulin-dependent protein kinase II α subunit
EPL: external plexiform layer
ETC: external tufted cell
5T4 (Tpbg): 5T4 oncofetal trophoblast glycoprotein in rodents
GABA: *γ*-aminobutyric acid
GC: granule cell
IPL: inner plexiform layer
KO: knockout
LRR: leucine-rich repeat
LV: lateral ventricle
MC: mitral cell
mGluR2: metabotropic glutamate receptor 2
OB: olfactory bulb
OPL: outer plexiform layer
OSN: olfactory sensory neurons
PKCα: protein kinase Cα
RBC: rod bipolar cell
RGC: retinal ganglion cell.
